# Resection of intrahepatic cholangiocarcinoma in octogenarians: a single-center analysis

**DOI:** 10.1007/s12672-024-01065-2

**Published:** 2024-06-12

**Authors:** Clara A. Weigle, Oliver Beetz, Bengt A. Wiemann, Philipp Tessmer, Simon Störzer, Sebastian Cammann, Florian W. R. Vondran, Felix Oldhafer, Moritz Schmelzle, Nicolas Richter

**Affiliations:** Department of General, Visceral and Transplant Surgery, Hannover Medical School, Carl-Neuberg-Str. 1, 30625 Hannover, Germany

**Keywords:** Intrahepatic cholangiocarcinoma, Resection of intrahepatic cholangiocarcinoma, Liver resection, Clavien-Dindo classification, Elderly patients, Octogenarians

## Abstract

The rapidly aging population in industrialized countries comes with an increased incidence of intrahepatic cholangiocarcinoma (iCC) which presents new challenges for oncological treatments especially in elderly patients. Thus, the question arises to what extent the benefit of surgical resections, as the only curative treatment option, outweighs possible perioperative risks in patients ≥ 80 years of age (octogenarians). We therefore retrospectively analyzed 311 patients who underwent resection for iCC at Hannover Medical School between January 1996 and December 2022. In total, there were 11 patients older than 80 years in our collective. Despite similar tumor size, octogenarians underwent comparatively less extensive surgery (54.5% major resections in octogenarians vs. 82.7% in all other patients; p = 0.033) with comparable rates of lymphadenectomy and tumor-free resection margins. Furthermore, we did not observe increased major postoperative morbidity (Clavien-Dindo ≥ IIIa complications: 27.3% vs. 34.3% in all other patients; p = 0.754) or mortality (estimated 1-year OS of 70.7% vs. 72.5% in all other patients, p = 0.099). The length of intensive care unit (ICU) or intermediate care unit (IMC) stay was significantly longer in octogenarians, however, with a comparable length in total hospital stay. The estimated overall survival (OS) did also not differ significantly, although a trend towards reduced long-term survival was observed (14.5 months vs. 28.03 months in all other patients; p = 0.099). In conclusion, primary resection is a justifiable and safe therapeutic option even in octogenarians but requires an even more thorough preoperative patient selection.

## Introduction

The declining birth rate combined with an increase in life expectancy has shifted the demographic structure in Germany, and most other industrialized countries, to a previously unknown extent. Analyses by the Federal Statistical Office of Germany show that every 5th person in Germany is now older than 65 years of age [1]. At the same time the incidence of primary liver tumors is increasing in Europe and North America [2]. This, combined with an aging population, poses new challenges for oncological treatments. Nanashima et al. showed in a Japanese cohort that 50% of liver resections for hepatocellular carcinoma are performed in patients older than 70 years. The authors attributed this to a significant increase in this age population already 5 years prior to the publication of these data in 2011 [3].

Tumors of the hepatobiliary system are among the most aggressive tumors with a 5-year OS of around 30% [4]. Currently, surgical resection remains the only curative option and is therefore regarded as first-line therapy. However, only 20–30% of cases are resectable at the time of diagnosis [5]. Moreover, resections are also associated with significant morbidity and mortality: A large German study from Filmann et al. reports hospital mortality rates of 11.0% after liver resections for iCC´s which increase to 16.2% when extended resections are performed and to 25% in case a reconstruction by biliodigestive anastomosis is required [6]. On the other hand, systemic therapy concepts have been defined recently: The introduction of durvalumab (PDL1 Inhibitor) showed promising results with significant prolongation of OS survival [7]. Therefore, it is already included in the current guidelines of the European Society for Medical Oncology (ESMO) as a first line therapy for advanced or metastatic biliary tract cancer and is currently being considered for adjuvant therapy [8].

This raises the question whether the significant perioperative risk outweighs the oncological benefit of liver resections, especially in vulnerable patient populations with reduced life expectancy. As an answer to this problem, general scoring indices such as the Charlson Comorbidity Index (CCI) were developed, predicting long-term mortality based on nineteen different underlying diseases. Especially in oncology, a modification of the CCI is used with the addition of patient age [9]. However, these general assessment tools have their limitations and do not provide a universal basis for oncological therapy decisions, especially considering the variability of the respective tumor biologies. Numerous studies therefore specifically examined at what age patients with carcinomas of the hepatobiliary system no longer benefit from surgical resections. Of note, most studies set the cut-off at 60 [10–12] or 70 years, respectively [13–15]. Considering that patients in Germany with 65 years of age still have a mean life expectancy of 17.8 years in men and 21.0 years in women, it seems unreasonable to not explore the only curative approach, especially since we are dealing with increasingly healthy elderly patients [16]. Therefore, we chose an age cut-off of 80 years for this retrospective single center analysis of 311 cases and investigated to what extent patients older than 80 years (octogenarians) benefit from primary resection.

## Materials and methods

### Study design

**Fig. 1:**
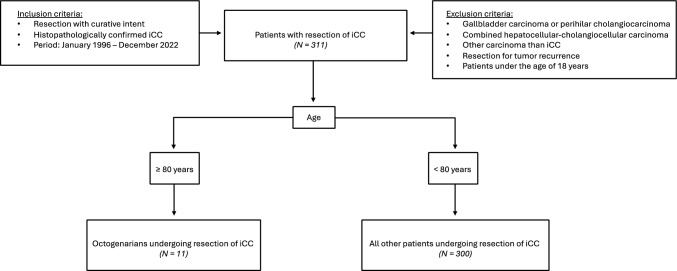
Schematic overview of all 311 patients included in our analysis. Of these N = 11 were octogenarian, N = 300 were under the age of 80

We retrospectively analyzed 311 patients undergoing resection with curative intent for histopathologically confirmed iCC at our institution from February 1996 until December 2022. For statistical analysis, we collected patient biometrics, preoperative laboratory data, surgical data, postoperative course and long-term survival. Patients with gallbladder-carcinoma, combined hepatocellular-cholangiocarcinoma and patients with perihilar cholangiocarcinoma were excluded from further analysis. Patients undergoing resection for tumor recurrence and pediatric patients (under 18 years of age) were also excluded. Our study design is shown schematically as a patient flow chart in Fig. 1.

### Definition of variables

Patients ≥ 80 years of age were defined as octogenarians. Preoperative anemia was defined as hemoglobin concentration lower than 13.5 g/dl in male patients and 11.5 g/dl in female patients. Preoperative leukocytosis was defined as a leukocyte concentration above 11,000 leukocytes/µl.

Based on the Brisbane classification, major hepatectomies were defined as resection of ≥ 3 liver segments, extended hepatectomies as resection of ≥ 5 liver segments [17]. Vascular resection was defined as additional resection of large vessels (e.g., portal vein or inferior vena cava). Multivisceral resection was defined as resection of extrahepatic tissue except for hiliary bile duct or large vessels which were recorded separately. Postoperative complications were coded according to the Clavien-Dindo classification. Severe complications were defined as Clavien-Dindo ≥ grade IIIa. Typical complication following liver surgery such as hemorrhage, biliary leakage and acute liver failure requiring intervention were classified as grade C complications according to the International Study Group of Liver Surgery (ISGLS) [18–20]. The current 8th edition of the American Joint Committee (AJCC) and the Union for International Cancer Control (UICC) edition was applied for the classification of iCC [21]. To avoid retrospective misclassification, patients were only classified if corresponding pathological parameters were available.

Follow-up time was defined as the time between day of surgery and the last contact or death. Survival times were reported as Kaplan–Meier median estimates.

### Statistical analysis

In case of normal distribution metric variables between the two groups were compared using Student’s t-test, otherwise the Mann–Whitney-U-test was applied. Categorial variables were compared using the Chi^2^-test or the Fisher-Exact-test, respectively. For comparison of survival data, the log-rank test was used. Univariate binary logistic regression analysis was performed to quantify the effect of the variables on nominal study endpoint. To analyze the effect of the variables on patient survival, we used univariate cox regression analysis. Multivariate regression analysis for identification of independent risk factors was performed, by a stepwise forward selection of purposeful selected variables with a p-value of < 0.300 in the univariate regression analysis and with missing values of < 10%. Statistical significance was set at a p-value of < 0.050 and is shown in bold (tables) or marked with an asterisk (figures). The collected data was analyzed using SPSS statistical software (version 28; SPSS Inc.; IBM Corporation, Armonk, NY, USA).

### Ethics

Within the general policy of our institution, patients or their legal guardians provided informed consent that their data may be used for scientific purpose. The ethical committee at Hannover Medical School stated that no further approval for retrospective data collection is needed. Prior analysis patient data and records were anonymized and de-identified. We confirm that all methods of our study were conducted in accordance with the relevant ethical guidelines and general regulations.

## Results

### Patient cohort

A total of 311 patients undergoing resection for histopathologically confirmed iCC were included in our study. Of these, 11 patients were octogenarians (80–84 years of age), whereas 300 patients were under the age of 80. None of the octogenarians showed signs for impaired liver function as defined by Quick with a mean of 97.96% (73.0–130.0%) and by platelet count with a mean of 306 × 10^3^/µl (152–478 × 10^3^/µl). Preoperative bilirubin levels were also even lower by trend (mean of 7.91 µmol/l vs. 21.3 µmol/l in all other patients; p = 0.119). Renal function (measured by preoperative creatinine levels) was also within normal levels, given the advanced age of the octogenarian patients. Octogenarians had significantly lower preoperative hemoglobin levels (mean of 12.18 g/dl vs. 13.29 g/dl in all other patients: p = 0.038).

### Surgical details

The analyzed data suggests that octogenarians underwent less extensive surgery: Even though comparable tumor sizes were seen in the patient collectives (7.6 cm in octogenarians vs. 6.9 cm in all other patients; p = 0.468), octogenarians underwent significantly fewer major hepatectomies and fewer extended hepatectomies by trend (54.5% and 18.2% vs. 82.7% and 36.0% in all other patients; p = 0.033 and p = 0.339). Consequently, significantly shorter surgery times were recorded for this patient cohort (105 min vs. 190 min in all other patients, p = 0.011). Despite a more parenchyma-sparing approach, intraoperative transfusions were required more frequently in octogenarians (63.6% vs. 46.0% in all other patients; p = 0.361). However, the number of packed red blood cells (PRBCs) transfused did not differ, suggesting low-threshold transfusion most likely in the presence of lower preoperative/baseline hemoglobin and reduced compensatory capacity. There were no limitations regarding the use of the Pringle maneuver, in fact we observed a trend towards a more frequent use in octogenarians (90.9% vs. 77.0% in all other patients; p = 0.370) whereas the cumulative occlusion time did not differ significantly (17 min vs. 21 min in all other patients; p = 0.306). Only two octogenarians underwent bile duct and vascular resection. None of the octogenarians underwent simultaneous extrahepatic resection (vs. 6.0% in all other patients; p = n.a.). All eleven patients analyzed underwent open surgical resection.

### Histopathological results

Despite the seemingly more cautious surgical approach, an equally adequate oncologic outcome was achieved in the octogenarian patients. There were no statistically significant differences in the rate of tumor-free resection margins (81.8% vs. 81.7% in all other patients; p = 1.000). There were also no differences in the rate of performed lymphadenectomies (63.6% vs. 66.0% in all other patients; p = 1.000) and an even higher median number of lymph nodes were removed by trend (7 (1–13) in octogenarians vs. 3 (1–23) in all other patients; p = 0.113). Of note, the generally low rates of lymphadenectomies in this study are owed to the lack of data on the necessity of lymphadenectomies for iCC in the earlier observation period, which is underscored by a noticeable increase in performed lymphadenectomies in the past five years, since the recommendation for removal of at least 6 lymph nodes was included in the AJCC/UICC 8th edition [22].

Of note, a poor tumor differentiation, corresponding to a grading (G) of > 2, was observed more frequently in the octogenarian patients by trend (45.5% vs. 28.0% in all other patients; p = 0.309). Table 1 gives an overview of the biometric data of the patient cohort, preoperative laboratory values, surgical details, and histopathological results. Table 2 summarizes the histopathological results and recommendations of the interdisciplinary tumor board of the octogenarians.

**Table 1:** Comparison of perioperative variables of octogenarians and patients under the age of 80 undergoing resection of intrahepatic cholangiocarcinoma

Variables	Octogenarians (≥ 80 years; n = 11)			Patients < 80 years (n = 300)			*p*-value
	N (%)	Mean, Median (Range)	M.v. (N (%)	N (%)	Mean, Median (Range)	M.v. (N (%)	
Biometrics							
Age (in years)		81.64, 81 (80–84)	0 (0)		60.86, 62 (24–79)	0 (0)	** < 0.001**
Male	5 (45.5)		0 (0)	156 (52)		0 (0)	0.764
BMI (in kg/m^2^)		24.71, 24.69 (18–34)	0 (0)		25.84 25.39 (16–55)	5 (1.7)	0.413
Preoperative laboratory values							
Hemoglobin (in g/dl)		12.18, 11.5 (10–14.8)	0 (0)		13.29, 13.4 (7.3–18.1)	1 (0.3)	**0.038**
Leukocytes (× 10^3^/µl)		8.32; 8.6 (5.5–11.2)	0 (0)		8.29, 7.7 (1.7–24.1)	1 (0.3)	0.500
Platelets (× 10^3^/µl)		306.6; 295 (152–478)	0 (0)		277.4, 253 (69–902)	5 (1.7)	0.162
Quick (in %)		97.96, 103 (73–130)	0 (0)		99.3, 100 (46–147)	3 (1.0)	0.855
ASAT (in U/l)		54.91, 31 (12–263)	0 (0)		39.5, 31 (4–304)	7 (2.3)	0.735
Bilirubin (in µmol/l)		7.91, 8 (4–13)	0 (0)		21.3, 9 (3–445)	11 (3.7)	0.119
Creatinine (in µmol/l)		79, 85 (44–116)	0 (0)		70.2 66 (39–165)	6 (2.0)	0.092
Surgical details							
Year of surgery		2012.73; 2015 (2000–2022)	0 (0)		2008.92; 2009 (1996–2022)	0 (0)	0.116
Operating times (in min)		152.3, 105 (81–358)	1 (9.1)		204.6, 190 (67–780)	3 (1.0)	**0.011**
Hepatic pedicle clamping	10 (90.9)		1 (9.1)	231 (77.0)		25 (8.3)	0.370
Hepatic pedicle clamping (in min)		17.9, 17 (5–34)	1 (9.1)		22.5, 21 (0–110)	29 (9.7)	0.306
Lymphadenectomy	7 (63.6)		0 (0)		198 (66.0)	0 (0)	1.000
Total number of lymph nodes		6.71, 7 (1–13)	4 (36.4)		4.9, 3 (1–23)	101 (33.7)	0.113
Major hepatectomy	6 (54.5)		0 (0)	248 (82.7)		0 (0)	**0.033**
Extended hepatectomy	2 (18.2)		0 (0)	108 (36.0)		0 (0)	0.339
Perihilar bile duct resection	2 (18.2)		0 (0)	58 (19.3)		0 (0)	1.000
Vascular resection	2 (18.2)		0 (0)	15 (5.0)		0 (0)	0.116
Extrahepatic resection	0 (0)		0 (0)	18 (6.0)		0 (0)	n.a
Intraoperative transfusion	7 (63.6)		0 (0)	138 (46.0)		6 (2.0)	0.361
Intraoperative units of PRBC		1.82, 1 (0–7)	0 (0)		1.95, 0 (0–17)	0 (0)	0.655
Histopathological details							
Tumor size (in cm)		8.51, 7.6 (4.7–21.0)	0 (0)		7.4, 6.9 (0.5–20.5)	2 (0.7)	0.468
Multifocality	3 (27.3)		0 (0)	107 (35.7)		0 (0)	0.752
Vascular invasion	2 (18.2)		0 (0)	27 (9.0)		28 (9.3)	n.a
N1	3 (27.3)		4 (36.4)	86 (28.7)		102 (34.0)	1.000
M1	0 (0)		0 (0)	10 (3.3)		1 (0.3)	n.a
G > 2	5 (45.5)		0 (0)	84 (28.0)		6 (2.0)	0.309
R1 und R2	2 (18.2)		0 (0)	52 (17.3)		3 (1.0)	1.000

Bold values indicate statistical significance

*M.v.* missing values, *BMI* body mass index, *ASAT* aspartate transaminase, *PRBC* packed red blood cells

**Table 2:** Oncological data of octogenarian patients

Patient	Year of surgery	Histology	UICC	Multifocal	Bilobar	Extrahepatic Resection	Vascular Resection	Recommendation for postoperative Chemotherapy
1	2000	T1b (10 cm), N0 (0/4), M0, Vx, G2, R0	1b	No	No	No	No	No
2	2004	T1b (7.5 cm), Nx, M0, Vx, G2, R0	n.a	No	No	No	No	No
3	2005	T2 (21 cm), N1 (1/7), M0, Vx, G3, R0	3b	Yes	No	no	No	No
4	2008	T2 (8.5 cm), N1 (3/8), M0, Vx, G3, R0	3b	Yes	No	No	No	Gemcitabine
5	2015	T1b (9 cm), Nx, M0, V0, G2, R0	n.a	No	No	No	No	No
6	2015	T1b, (7.6 cm), N0 (0/7), M0, V0, G2, R1	1b	No	Yes	No	Yes	No
7	2016	T1a (4.7 cm), N1 (3/7), M0, Vx, G3, R0	3b	No	No	No	No	Gemcitabine and cisplatin
8	2017	T1b (8.5 cm), Nx, M0, V0, G3, R0	n.a	No	No	No	No	Capecitabine
9	2018	T1b (5.2 cm), N0 (0/1), M0, V0, R0	1b	No	No	No	No	Capecitabine
10	2020	T1a (4.9 cm), Nx, M0, V0, G3, R0	1a	No	No	No	No	No
11	2022	T2 (6.7 cm), N0 (0/13), M0, Vx, G2, R1	2	Yes	Yes	No	No	Capecitabine

*UICC* Union for International Cancer Control; *n.a.* not applicable/not applied

### Postoperative course and survival

Despite their high age, octogenarians displayed lower rates of severe postoperative complications defined as Clavien-Dindo ≥ IIIa by trend (27.3% vs. 34.3% in all other patients; p = 0.754). Postoperative transfusion requirements were also lower by trend (18.2% vs. 28.2% all other patients; p = 0.729). One patient developed biliary leakage, which could be addressed interventionally and another patient developed respiratory failure, which required prolonged intensive medical treatment. A third octogenarian patient showed post hepatectomy liver failure (PHLF) after trisegmentectomy with bile duct resection and vascular reconstruction and died in the postoperative course due to a combined pulmonary failure after medical history of coronary artery disease and chronic obstructive pulmonary syndrome leading to a multi-organ-failure. There was no significant difference regarding intrahospital mortality (9.1% in octogenarian vs. 6.7% in all other patients, p = 0.543). Octogenarian patients had significantly longer postoperative ICU/IMC stays (3 (1–47) days vs. 2 (0–91) days in all other patients; p = 0.041). However, the overall hospital stay did not differ significantly (19 (9–48) days 20 (4–95) days in all other patients, p = 0.739). Furthermore, the estimated 1-year OS showed no significant differences (70.7% vs. 72.5% in all other patients; p = n.a.). However, there was a trend towards a reduced estimated 3- and 5-year OS, which must be put into perspective by the naturally reduced life expectancy of octogenarians (11.8% after 3 years and 0% after 5 years in octogenarians vs. 42.7% and 27.6% in all other patients; p = n.a.). The estimated OS of octogenarians was accordingly lower, although not statistically significant (14.5 months vs. 28.03 months in all other patients; p = 0.099).

Regarding the further oncologic care of octogenarians, the recommendations of the interdisciplinary tumor board were vague. The possibility of an adjuvant therapy was frequently alluded, although without a clear recommendation due to the advanced age and a lack of data.

**Fig. 2:**
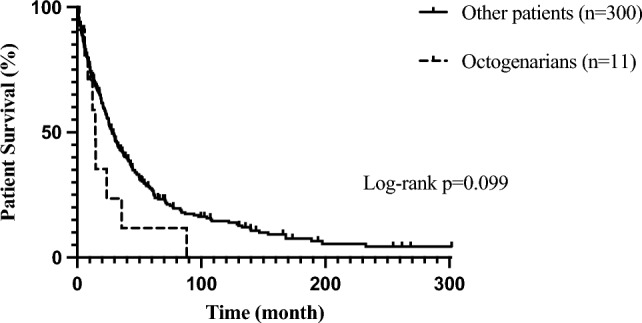
Kaplan–Meier analysis of overall survival of octogenarians (n = 11) and all other patients < 80 years of age (n = 300). No significant differences were detected

Figure 2 shows the estimated OS of both groups in Kaplan–Meier analysis. Table 3 gives an overview of the postoperative course, survival, and long-term OS of both groups. Table 4 shows the extent of surgery and the postoperative course of all octogenarians.

**Table 3:** Comparison of postoperative course and survival after resection of intrahepatic cholangiocarcinoma

Variables	Octogenarians (> 80 years; n = 11)			Younger patients (< 80 years; n = 300)			*p*-value
	N (%)	Mean, Median (Range)	M.v. (N (%)	N (%)	Mean, Median Range	M.v. (N (%)	
Postoperative course							
Postoperative transfusion (yes)	2 (18.2)		1 (9.1)	86 (28.7)		16 (5.3)	0.729
Postoperative units of PRBC		1.8, 0, (0–16)	1 (9.1)		1.5, 0 (0–29)	16 (5.3)	0.544
Postoperative complications ≥ CD IIIa	3 (27.3)			103 (34.3)		3 (1.0)	0.754
Biliary leakage ISGLS Grade C	1 (9.1)			14 (4.7)		2 (0.7)	0.427
Hemorrhage ISGLS Grade C	0 (0)			13 (4.3)		2 (0.7)	n.a
PHLF ISGLS Grade C	1 (9.1)			16 (5.3)		2 (0.7)	0.469
ICU stay (in days)		8.55, 3 (1–47)	0 (0)		4.52, 2 (0–91)	0 (0)	**0.041**
Hospital stay (in days)		21.18, 19 (9–48)	0 (0)		23.17, 20 (4–95)	0 (0)	0.739
Intrahospital mortality	1 (9.1)		0 (0)	20 (6.7)			0.543
30-day mortality	0 (0)		0 (0)	13 (4.3)		8 (2.7)	n.a
90-day mortality	1 (9.1)		0 (0)	24 (8.0)		11 (3.7)	1.000
Postoperative survival							
Follow-up (in months)		20.12, 12.39 (1.54–88.18)	0 (0)		38.49, 22.19 (0.10–301.74)	0 (0)	0.221
Dead at time of last follow-up	9 (81.8)		0 (0)	226 (75.3)		0 (0)	1.000
KM Survival		23.93, 14.52 (n.a.)	0 (0)		55.38, 28.03 (n.a.)		0.099
KM 1-year survival (in %)	70.7		0 (0)	72.5		0 (0)	
KM 3-year survival (in %)	11.8		0 (0)	42.7		0 (0)	
KM 5-year survival (in %)	0		0 (0)	27.6		0 (0)	

Bold values indicate statistical significance

*PRBC* Packed red blood cells, *CD* Clavien-Dindo, *ISGLS* International Study Group of Liver Surgery, *KM* Kaplan–Meier estimate, *n.a.* not applicable/not applied

**Table 4:** Surgical detail and postoperative course of octogenarian patients

Patient	Age	Underlying diseases	Operation	Operation time (minutes)	ICU stay (days)	Postoperative complications (CD)	Deceased at the time of last contact (survival in months)
1	80	None	Left hemihepatectomy, lymphadenectomy	170	3	2	Yes (88.18)
2	81	None	Left hemihepatectomy	81	3	0	Yes (12.39)
3	83	Cardiac, thromboembolic	Left hemihepatectomy, lymphadenectomy	107	7	0	Yes (5.88)
4	81	Cardiac, pulmonary	Left hemihepatectomy, lymphadenectomy	308	3	3b (bile leakage)	Yes (14.52)
5	83	Cardiac, thromboembolic	Atypical resection	89	5	2	Yes (8.41)
6	84	Cardiac, pulmonary	Extended hemihepatectomy right, lymphadenectomy, bile duct and vascular resection	358	47	5 (FLR failure)	Yes (intrahospital death)
7	81	Cardiac	Atypical resection, lymphadenectomy	105	3	4a (pulmonary failure)	Yes (35.88)
8	82	None	Atypical resection	92	3	0	Yes (23.75)
9	80	Cardiac, pulmonary	Segmental resection right, lymphadenectomy	99	4	0	Yes (14.88)
10	83	Thromboembolic	Segmental resection right	104	15	2	No (10.48)
11	80	None	Extended hemihepatectomy left, lymphadenectomy	162	1	1	No (5.39)

*ICU* Intensive care unit, *CD* Clavien-Dindo, *FLR* functional liver remnant thromboembolic previous thrombotic or embolic events

The results of univariate binary logistic regression analysis for identification of risk factors for the onset of severe postoperative complications defined as ≥ Clavien-Dindo IIIa are provided in Table 5. Multivariate analysis revealed BMI (OR 1.061; CI_95%_ 1.002–1.124; p = 0.042), the number of intraoperative PRBCs (OR: 1.182; CI_95%_ 1.076–1.299; p < 0.001) and the simultaneous resection of the perihilar bile ducts (OR: 3.545; CI_95%_ 1.829–6.871; p < 0.001) as independent risk factors for severe postoperative complications.

**Table 5:** Uni- and multivariable regression analysis for identification of risk factors for the onset of postoperative complications (≥ Clavien-Dindo IIIa) within the postoperative course after resection of intrahepatic cholangiocarcinoma

Variables	Univariable regression analysis			Multivariable regression analysis		
	OR	CI_95%_	*p*-value	OR	CI_95%_	*p-*value
Biometrical data						
Octogenarians	0.706	[0.183–2.720]	0.613			
Age (in years)	0.988	[0.968–1.009]	0.259			
Male	1.161	[0.725–1.861]	0.534			
BMI (in kg/m^2^)	1.074	[1.019–1.132]	**0.007**	1.061	[1.002–1.124]	**0.042**
PSC	1.313	[0.362–4.761]	0.678			
Colitis	1.496	[0.565–3.963]	0.418			
Nicotine	0.931	[0.458–1.891]	0.843			
Preoperative laboratory values						
Hemoglobin (in g/dl)	0.915	[0.800–1.046]	0.194			
Leukocytes (× 10^3^/µl)	1.045	[0.967–1.128]	0.267			
Platelets (× 10^3^/µl)	1.000	[0.998–1.003]	0.725			
Quick (in %)	0.983	[0.968–0.998]	**0.024**			
ASAT (in U/l)	1.009	[1.002–1.016]	**0.011**			
Bilirubin (in µmol/l)	1.008	[1.001–1.015]	**0.017**			
Creatinine (in µmol/l)	1.000	[0.987–1.014]	0.971			
Surgical details						
Year of surgery	1.001	[0.970–1.032]	**0.974**			
Operating times (in min)	1.006	[1.003–1.009]	**<0.001**			
Hepatic pedicle clamping	0.975	[0.493–1.928]	0.942			
Hepatic pedicle clamping (in min)	1.007	[0.992–1.023]	0.340			
Lymphadenectomy	1.123	[0.681–1.852]	0.650			
Total number of lymph nodes	1.054	[0.987–1.125]	0.117			
Major hepatectomy	2.547	[1.258–5.157]	**0.009**			
Extended hepatectomy	1.439	[0.884–2.342]	0.144			
Perihilar bile duct resection	3.490	[1.949–6.249]	** < 0.001**	3.545	[1.829–6.871]	** < 0.001**
Vascular resection	2.902	[1.071–7.859]	**0.036**			
Extrahepatic resection	1.228	[0.462–3.265]	0.681			
Intraoperative transfusion	1.983	[1.225–3.208]	**0.005**			
Intraoperative units of PRBC	1.188	[1.088–1.298]	** < 0.001**	1.182	[1.076–1.299]	** < 0.001**
Histopathological details						
Tumor size (in cm)	1.028	[0.966–1.094]	0.384			
Multifocality	1.496	[0.920–2.432]	0.105			
Vascular invasion	1.813	[0.816–4.031]	0.144			
N1	1.253	[0.703–2.232]	0.445			
M1	0.466	[0.097–2.236]	0.340			
G > 2	1.455	[0.871–2.429]	0.152			
R1 und R2	1.920	[1.052–3.501]	**0.033**			

Bold values indicate statistical significance

The results of univariate cox regression analysis to quantify the effect of variables on patient survival are shown in Table 6. Multivariate cox regression analysis revealed regular nicotine consumption (HR: 2.032; CI_95%_ 1.267–3.258; p = 0.042), preoperative leukocytosis (HR: 1.064; CI_95%_ 1.005–1.125; p = 0.032), number of intraoperative PRBCs (HR: 1.094; CI_95%_ 1.024–1.170; p = 0.008) and N1 status (HR: 1.879; CI_95%_ 1.325–2.664; p < 0.001) as independent risk factors for patient survival.

**Table 6:** Uni- and multivariable regression analysis for identification of risk factors patient survival after resection of intrahepatic cholangiocarcinoma

Variables	Univariable regression analysis			Multivariable regression analysis		
	HR	CI_95%_	*p*-value	HR	CI_95%_	*p-*value
Biometrical data						
Octogenarians	1.742	[0.892–3.403]	0.104			
Age (in years)	0.999	[0.987–1.011]	0.889			
Male	0.953	[0.737–1.231]	0.711			
BMI (in kg/m^2^)	1.018	[0.990–1.048]	0.213			
PSC	0.956	[0.393–2.323]	0.921			
Colitis	1.040	[0.571–1.895]	0.897			
Nicotine	1.579	[1.084–2.301]	**0.017**	2.032	[1.267–3.258]	**0.003**
Preoperative laboratory values						
Hemoglobin (in g/dl)	0.902	[0.835–0.974]	**0.009**			
Leukocytes (× 10^3^/µl)	1.090	[1.048–1.133]	** < 0.001**	1.064	[1.005–1.125]	**0.032**
Platelets (× 10^3^/µl)	1.001	[1.000–1.002]	**0.046**			
Quick (in %)	0.984	[0.975–0.993]	** < 0.001**			
ASAT (in U/l)	1.005	[1.001–1.008]	**0.015**			
Bilirubin (in µmol/l)	1.004	[1.002–1.007]	** < 0.001**			
Creatinine (in µmol/l)	1.003	[0.996–1.011]	0.427			
Surgical details						
Year of surgery	1.000	[0.980–1.020]	0.998			
Operating times (in min)	1.002	[1.001–1.004]	** < 0.001**			
Hepatic pedicle clamping	0.833	[0.574–1.207]	0.333			
Hepatic pedicle clamping (in min)	1.001	[0.992–1.010]	0.856			
Lymphadenectomy	1.377	[1.048–1.809]	**0.022**			
Total number of lymph nodes	1.023	[0.990–1.057]	0.176			
Major hepatectomy	1.080	[0.765–1.525]	0.661			
Extended hepatectomy	1.150	[0.881–1.500]	0.305			
Perihilar bile duct resection	1.485	[1.081–2.039]	**0.015**			
Vascular resection	1.879	[1.041–3.393]	**0.036**			
Extrahepatic resection	3.134	[1.718–5.719]	** < 0.001**			
Intraoperative transfusion	1.558	[1.202–2.018]	** < 0.001**			
Intraoperative units of PRBC	1.094	[1.048–1.141]	** < 0.001**	1.094	[1.024–1.170]	**0.008**
Histopathological details						
Tumor size (in cm)	1.032	[1.000–1.066]	**0.050**			
Multifocality	1.446	[1.109–1.884]	**0.006**			
Vascular invasion	1.458	[0.952–2.232]	0.083			
N1	1.971	[1.421–2.735]	** < 0.001**	1.879	[1.325–2.664]	** < 0.001**
M1	2.173	[0.958–4.926]	0.063			
G > 2	1.154	[0.872–1.528]	0.316			
R1 und R2	1.139	[0.812–1.598]	0.450			
Postoperative course						
Postoperative transfusion (yes)	1.349	[1.015–1.792]	**0.039**			
Postoperative units of PRBC	1.175	[1.132–1.220]	** < 0.001**			
Postoperative complications ≥ CD IIIa	1.762	[1.350–2.298]	** < 0.001**			
Biliary leakage ISGLS Grade C	0.807	[0.451–1.445]	0.471			
Hemorrhage ISGLS Grade C	1.870	[0.991–3.528]	0.053			
PHLF ISGLS Grade C	10.934	[6.260–19.099]	** < 0.001**			
ICU stay (in days)	1.039	[1.025–1.052]	** < 0.001**			
Hospital stay (in days)	1.015	[1.005–1.026]	**0.004**			

Bold values indicate statistical significance

## Discussion

Although only 20–30% of patients with iCC can be considered for resection at the time of diagnosis, it remains to be the only curative approach for this tumor entity [5]. Despite this, liver resections are associated with significant morbidity and mortality rates in the postoperative course [6]. With an increasingly aging society, the fraction of elderly patients with iCC is rising as several studies suggest [23, 24]. Thus, the question how to proceed with patients of advanced age suffering from iCC is pressing. Only few studies specifically focus on very elderly patients suffering iCC: Vitale et al. showed in a multicenter analysis that patients ≥ 70 years of age had significantly more minor as well as major complications with, however, comparable 30- and 90-day mortality rates [25]. Contrary to this, Bartsch et al. showed that there were no differences regarding resectability, resection extent and perioperative morbidity as well as mortality [24]. In line with Bartsch et al., we could not demonstrate significant differences in postoperative complication rates between the octogenarians and all other patients in our retrospective analysis of 311 patients undergoing resection for iCC. Other than the afore-mentioned study, we observed significantly lower rates of major resections in octogenarian patients, reflecting a more parenchyma-sparing approach possibly due to the preoperative patient selection. Even though other authors described significant decreased rates of tumor-free resection margins and thus a reduced DFS in patients > 70 years of age suffering from colorectal liver metastases [26], likely due to a more parenchyma sparing approach, we could not observe different R0 resection rates in our octogenarian patients. Due to the scarce data on iCC in octogenarian patients, we need to include the data on liver resections for other tumor entities:

Confirming the observations we made in our patient collective, several studies analyzing the outcomes of octogenarians after resection of perihilar cholangiocarcinoma demonstrated comparable postoperative complication rates in octogenarians [27, 28]. Also, for resections of colorectal liver metastases, comparable perioperative outcomes in elderly patients are described, thus being in line with the afore-mentioned literature and our results [29]. In contrast to this, some authors describe significantly increased rates of major complications and consequently significantly increased mortality after resection of malignant hepatic tumors in elderly patients [14, 15]. However, this seems to be mainly due to higher rates of pneumonia, renal failure and infectious diseases. The liver-surgery associated complications rates did not differ even in elderly patients [13]. A study evaluating risk factors for postoperative complications after resection of hepatocellular carcinoma was able to demonstrate that age plays a substantial role, most likely as a result of underlying age-associated comorbidities [30], also noted for liver resections in general [15]. In contrast, our subgroup of octogenarians displayed better renal and liver function as well as lower baseline bilirubin levels compared to younger patients. This, in addition to the fact that only eleven octogenarians underwent resection for iCC over a 26-year observation period, strongly suggests a substantial preoperative patient selection which could pose a reason for comparable complication rates between octogenarians and all other patients.

A more rigid preoperative selection seems to play a key role in offering a safe therapeutic approach to elderly patients. This selection is usually done in interdisciplinary tumor boards, considering liver function parameters, prior and present comorbidities, the planned extent of resection and the intuition of experienced hepatobiliary surgeons. Additionally, to evaluate the operability of elderly patients on a more objectifiable basis, general assessments such as the American society of Anesthesiologist (ASA) score, the Eastern cooperative oncology group (ECOG) score or the CCI are usually applied [31–33]. Although some authors showed that an ASA score of ≥ 3 is a risk factor for a complicative course after resection of colorectal liver metastases in patients > 75 years and others postulated that a high CCI is an independent risk factor for postoperative complications following resection of hepatocellular carcinoma, conventionally applied scoring systems are limited by their non-disease- or non-surgery-specific character [29, 30]. Starlinger et al. recently published that a combination of the aspartate aminotransferase/platelet ratio index (APRI) and the albumin bilirubin grade (ALBI) bears a high predictive potential with regards to perioperative mortality, albeit without acknowledging age as an important factor [34]. For example, Tanaka et al. used a prospective multicenter study to develop the “frailty index”, which can predict age-adjusted complications (e.g., delirium, cardiopulmonary outcomes and increased need for rehabilitation) after liver resection [35]. A combination of disease-specific and age-specific scores may hold the potential for adequate decision making in high-risk groups such as octogenarians in the future.

In addition to appropriate patient selection based on the parameters shown above, prehabilitation and enhanced recovery after surgery (ERAS) concepts possess the potential to enable safe surgery especially for elderly patients [36].

Other than a careful patient selection and above-mentioned prehabilitation concepts, the surgical procedure of choice influences the postoperative outcome. While minimally invasive liver surgery is increasingly performed and has become the clinical standard in hepatobiliary centers, cholangiocarcinoma is often regarded as the last bastion for open surgery, hence all patients in our analysis underwent open surgical resection. Nevertheless, a growing number of authors have been able to publish promising results for minimally invasive approaches (including robotic-assisted surgery [37–39]) for liver resection, demonstrating lower morbidity and shorter hospital length of stay, without inferior oncological outcomes. Therefore, especially elderly patients could benefit significantly from minimally invasive resection of iCC [40–42].

Current available data focusing on age as a potential risk factor after liver resection lack differentiation between different tumor entities [11–13, 15, 43, 44], although this distinction is necessary when providing treatment recommendations based on age-specific complications in relation to the oncological benefit of surgical resections. Recently, an analysis based on the SEER database was published by Chen et al. which compared a primary surgical approach by resection with a non-surgical therapy (chemotherapy, radiation) in resectable patients > 60 years of age with histologically confirmed iCC. This study shows clear oncologic benefit for patients undergoing resection even in case of pre-existing lymph node metastases [10]. However, the cut-off defining “elderly patients” seems disputable considering increasing life expectancy. Above-mentioned study also included a subgroup analysis of octogenarians (with a total of 26 patients) in which the superiority of surgical resection was also shown [10]. Accordingly, our multivariate analysis did not reveal any age-related risk factors for severe postoperative complications or patient survival. Thus, when resectability and operability of patients are given, conservative approaches such as chemotherapy, radiation, or immunotherapy hardly pose an alternative. Vitale et al. could also show in the afore-mentioned multicenter study analyzing the resection of iCC in patients > 70 years of age that there is no difference in 5-year OS and DFS [25]. Consistent with the literature, our data indicate that octogenarians do not have a significantly shorter OS, although a trend towards inferior survival was observed. The latter fact may be partly due to the reduced average life expectancy of octogenarians in general with the average life expectancy of 81-year-olds (median age in our cohort) being 7.28 years in men and 8.51 years in women in Germany in 2009 (median time point of the analyzed period) according to official mortality tables [45] which raises questions regarding the benefit of adjuvant therapy. Although adjuvant therapy with capecitabine over 6 months is clearly recommended in the guidelines [46] the recommendations of the tumor board regarding adjuvant therapy in our collective were comparatively vague and restrictive most likely due to advanced age of these patients and lack of data. Due to carefully patient selection, we could observe comparable complication rates even after surgical resection, the feasibility of adjuvant chemotherapy should not be dismissed purely due to age.

A major limitation of our study is the retrospective nature, the missing data on disease-free survival and the long observation period spanning three decades. Although the number of cases analyzed is large compared to other single-center analyses, the statistical analysis of eleven octogenarians is not sufficient to draw definite consequences. Hence, multi-center analyses are required to provide strong evidence for therapeutic decision-making in this vulnerable patient collective.

## Conclusion

Our data indicate that resection of iCC is feasible and safe in octogenarians and is not associated with more severe complications in the postoperative course or limited overall survival. However, a meticulous preoperative patient selection based on biological and not on chronological age seems essential for a successful surgical approach. Further multicenter analyses are required to identify additional risk factors and to derive definitive consequences for this clinically relevant question.

## Data Availability

The datasets used and/or analyzed during the current study are available from the corresponding author on reasonable request.

## Abbreviations

AJCC: American Joint Committee
ALBI: Albumin bilirubin grade
APRI: Aspartate aminotransferase/platelet ratio index
ASA: American society of Anesthesiologist
CCI: Charlson comorbidity index
DFS: Disease free survival
ECOG: Eastern cooperative oncology group
ERAS: Enhanced recovery after surgery
ESMO: European society for medical oncology
iCC: Intrahepatic cholagniocarcinoma
ICU: Intense care unit
IMC: Intermediate care unit
ISGLS: International Study Group of Liver Surgery
OS: Overall survival
PHLF: Post hepatectomy liver failure
PRBC: Packed red blood cells
UICC: Union for International Cancer Control
